# CD32^+^ and PD-1^+^ Lymph Node CD4 T Cells Support Persistent HIV-1 Transcription in Treated Aviremic Individuals

**DOI:** 10.1128/JVI.00901-18

**Published:** 2018-09-26

**Authors:** Alessandra Noto, Francesco A. Procopio, Riddhima Banga, Madeleine Suffiotti, Jean-Marc Corpataux, Matthias Cavassini, Agostino Riva, Craig Fenwick, Raphael Gottardo, Matthieu Perreau, Giuseppe Pantaleo

**Affiliations:** a Service of Immunology and Allergy, Lausanne University Hospital, University of Lausanne, Lausanne, Switzerland; b Service of Vascular Surgery, Lausanne University Hospital, University of Lausanne, Lausanne, Switzerland; c Service of Infectious Diseases, Lausanne University Hospital, University of Lausanne, Lausanne, Switzerland; d Vaccine Division, Fred Hutchinson Cancer Research Center, Seattle, Washington, USA; e Infectious Disease Division, Fred Hutchinson Cancer Research Center, Seattle, Washington, USA; f Swiss Vaccine Research Institute, Lausanne University Hospital, University of Lausanne, Lausanne, Switzerland; g Infectious Disease Division, Luigi Sacco University Hospital, Milan, Italy; Emory University

**Keywords:** CD32, lymph node, PD-1, Tfh cells, human immunodeficiency virus

## Abstract

A recent study conducted in blood has proposed CD32 as the marker identifying the “elusive” HIV reservoir. We have investigated the distribution of CD32^+^ CD4 T cells in blood and lymph nodes (LNs) of HIV-1-uninfected subjects and viremic untreated and long-term-treated HIV-1-infected individuals and their relationship with PD-1^+^ CD4 T cells. The frequency of CD32^+^ CD4 T cells was increased in viremic compared to treated individuals in LNs, and a large proportion (up to 50%) of CD32^+^ cells coexpressed PD-1 and were enriched within T follicular helper (Tfh) cells. We next investigated the role of LN CD32^+^ CD4 T cells in the HIV reservoir. Total HIV DNA was enriched in CD32^+^ and PD-1^+^ CD4 T cells compared to CD32^−^ and PD-1^−^ cells in both viremic and treated individuals, but there was no difference between CD32^+^ and PD-1^+^ cells. There was no enrichment of latently infected cells with inducible HIV-1 in CD32^+^ versus PD-1^+^ cells in antiretroviral therapy (ART)-treated individuals. HIV-1 transcription was then analyzed in LN memory CD4 T cell populations sorted on the basis of CD32 and PD-1 expression. CD32^+^ PD-1^+^ CD4 T cells were significantly enriched in cell-associated HIV RNA compared to CD32^−^ PD-1^−^ (averages of 5.2-fold in treated individuals and 86.6-fold in viremics), CD32^+^ PD-1^−^ (2.2-fold in treated individuals and 4.3-fold in viremics), and CD32^−^ PD-1^+^ (2.2-fold in ART-treated individuals and 4.6-fold in viremics) cell populations. Similar levels of HIV-1 transcription were found in CD32^+^ PD-1^−^ and CD32^−^ PD-1^+^ CD4 T cells. Interestingly, the proportion of CD32^+^ and PD-1^+^ CD4 T cells negatively correlated with CD4 T cell counts and length of therapy. Therefore, the expression of CD32 identifies, independently of PD-1, a CD4 T cell population with persistent HIV-1 transcription and coexpression of CD32 and PD-1, the CD4 T cell population with the highest levels of HIV-1 transcription in both viremic and treated individuals.

**IMPORTANCE** The existence of long-lived latently infected resting memory CD4 T cells represents a major obstacle to the eradication of HIV infection. Identifying cell markers defining latently infected cells containing replication-competent virus is important in order to determine the mechanisms of HIV persistence and to develop novel therapeutic strategies to cure HIV infection. We provide evidence that PD-1 and CD32 may have a complementary role in better defining CD4 T cell populations infected with HIV-1. Furthermore, CD4 T cells coexpressing CD32 and PD-1 identify a CD4 T cell population with high levels of persistent HIV-1 transcription.

## Abstract (precis)

The existence of long-lived latently infected resting memory CD4 T cells represents a major obstacle to the eradication of HIV infection. Identifying cell markers defining latently infected cells containing replication-competent virus is important in order to determine the mechanisms of HIV persistence and to develop novel therapeutic strategies to cure HIV infection. We provide evidence that PD-1 and CD32 may have a complementary role in better defining CD4 T cell populations infected with HIV-1. Furthermore, CD4 T cells coexpressing CD32 and PD-1 identify a CD4 T cell population with high levels of persistent HIV-1 transcription.

## INTRODUCTION

The existence of long-lived latently HIV-1-infected resting memory CD4 T cells represents a major obstacle to the eradication of HIV infection (1–6). Several studies in recent years have greatly contributed to the characterization of different CD4 T cell populations containing inducible replication-competent HIV-1. In the blood, central memory CD4 T cells defined by the CD45RA^−^ CCR7^+^ CD27^+^ phenotype and transitional memory CD4 T cells defined by the CD45RA^−^ CCR7^−^ CD27^+^ phenotype have been identified as major cellular compartments of the latent HIV-1 reservoir, while CD4 T cells with stem cell-like properties have been identified as a minor component (7, 8). Recent studies performed in blood have also shown that a series of markers such as PD-1, LAG-3, HLA-DR, CCR6, TIGIT, and CD30 help to define CD4 T cell populations infected with HIV-1 (9–11). Studies performed in memory CD4 T cell populations isolated from lymph nodes (LNs) of viremic and long-term-treated HIV-1-infected individuals have demonstrated that PD-1-positive and T follicular helper (Tfh) CD4 T cells are the major reservoir for replication-competent and infectious virus in both viremic and long-term-treated individuals (12, 13). A recent study performed in blood has proposed that the low-affinity receptor for the immunoglobulin G Fc fragment, CD32a, defines a small population of HIV-1-infected resting CD4 T cells and therefore that CD32a^+^ CD4 T cells represent the elusive HIV reservoir (14). A recent study has challenged the findings that CD32a is a marker defining the elusive HIV reservoir (15). CD32^+^ CD4 T cells expressed several markers of activation, and they are not enriched in HIV DNA, while they are enriched for transcriptionally active HIV in long-term-treated individuals. Along the same line, another study performed in blood also failed to show enrichment of HIV-infected CD4 T cells within CD32^+^ cells (16). On the basis of the CD32a study and our previous observations on the role of PD-1^+^/Tfh cells in the HIV reservoir (12–14), we have addressed the following issues: (i) the differences in the percentages of CD32^+^ CD4 T cells in HIV-uninfected versus HIV-infected individuals; (ii) the distribution of CD32 in blood versus lymphoid tissue, i.e., LNs; (iii) the distribution of CD32 in different populations of memory CD4 T cells; and (iv) the relationship between CD32^+^ and PD-1^+^ CD4 T cell populations and their role in defining the HIV reservoir.

(This article was submitted to an online preprint archive [17].)

## RESULTS

### Distribution of CD32^+^ CD4 T cells in blood and lymph nodes.

**FIG 1:**
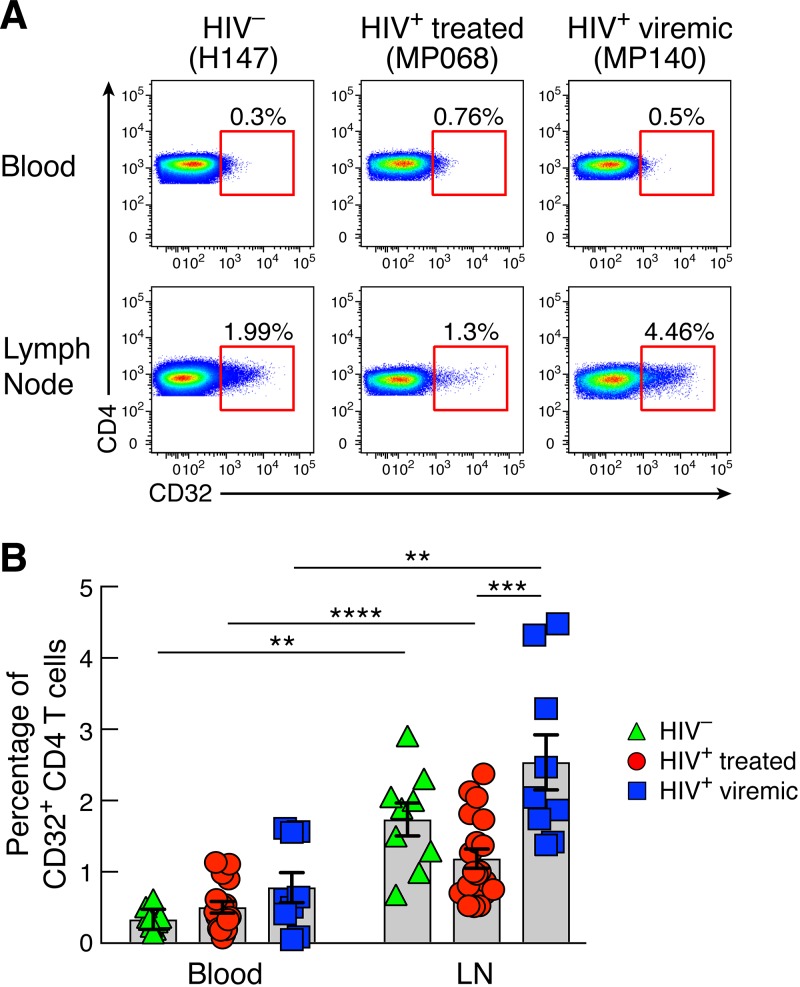
CD32 expression on CD4 T cells in LNs and blood of HIV-1-infected and uninfected individuals. LN and PB mononuclear cells were isolated from the same HIV-uninfected (*n* = 9), HIV-1-infected ART-treated (*n* = 19), and HIV-1-infected viremic (*n* = 9) individuals. Cells were stained with anti-CD3, anti-CD4, anti-CD45RA, and anti-CD32 antibodies. (A) Representative flow cytometry profiles of blood and LN memory (CD45RA^−^) CD4 T cell populations expressing CD32 in representative HIV-1-uninfected, ART-treated, and viremic subjects. (B) Cumulative data on the frequencies of CD32^+^ memory CD4 T cells in blood and LN mononuclear cells of HIV-1-uninfected, ART-treated, and viremic individuals. *P* values were obtained by a Mann-Whitney test to compare the three groups and a Wilcoxon signed-rank test to compare frequencies between blood and LNs. **, *P* < 0.01; ***, *P* < 0.001; ****, *P* < 0.0001. Error bars denote means ± standard errors of the means (SEM).

Blood and LN biopsy specimens were obtained from 9 HIV-uninfected subjects, 19 long-term antiretroviral therapy (ART)-treated individuals, and 9 viremic individuals (Table 1). In HIV-uninfected individuals, lymph node biopsy specimens were obtained from patients undergoing vascular surgery. A small percentage (<1%) of memory CD4 T cells in blood expressed CD32, but there were no significant differences between HIV-uninfected, long-term ART-treated, and viremic individuals (Fig. 1A and B). Memory CD4 T cells from LNs of the same individuals contained significantly higher percentages of CD32^+^ cells than in blood (*P* = 0.003 for HIV-uninfected individuals, *P* < 0.0001 for ART-treated individuals, and *P* = 0.003 for viremics). The percentage of LN CD32^+^ CD4 T cells was significantly higher in viremics than in long-term ART-treated individuals (Fig. 1A and B) (*P* = 0.0007) but not HIV-uninfected individuals (Fig. 1A and B).

**TABLE 1:** Study cohort: clinical data

Patient	Age (yr)	Sex^*a*^	Duration of HIV infection (yr)	Yr of suppressive ART	Viral load (copies/ml)	CD4 count (cells/μl)	ART status
MP106	37	M	0.1	0	160,000	501	Treatment naive
MP140	23	M	0.08	0	360,000	427	Treatment naive
MP119	23	M	1.3	0	13,000	498	Treatment naive
MP124	46	F	0.06	0	510,000	468	Treatment naive
MP117	51	M	5.42	0	54,000	504	Treatment naive
MP125	35	M	0.16	0	17,000	511	Treatment naive
MP118	29	M	0.06	0	14,000	538	Treatment naive
SA150	53	M	10	0	6,900	578	Treatment naive
SA143	37	M	0.3	0	25,000	704	Treatment naive
MP129	44	M	14.85	14.71	<20	928	ART treated
MP068-1	45	M	3.33	3	<20	928	ART treated
MP136	44	M	7.5	6	<20	732	ART treated
MP123	61	M	9.25	9	<20	1,270	ART treated
MP137	39	M	3.59	2.84	24	700	ART treated
MP064	36	M	7.88	4.49	<20	719	ART treated
MP141	31	M	11.56	1.5	<20	558	ART treated
MP094	47	M	8	8	<20	451	ART treated
MP058	46	F	10.86	10	<20	666	ART treated
MP082	53	M	22.45	8	<20	1,236	ART treated
MP096	54	F	18	13	<20	1,253	ART treated
MP103	41	M	1.62	1.16	<20	217	ART treated
MP068-2	47	M	5.12	5	<20	487	ART treated
MP060	42	M	21	8	<20	786	ART treated
MP067	41	M	14.01	14	<20	609	ART treated
MP072	47	M	18	2	<20	614	ART treated
MP070	55	M	7.06	2	<20	615	ART treated
MP100	36	F	13.06	10	<20	643	ART treated
MP145	39	M	8.35	4	<20	820	ART treated

a M, male; F, female.

These results indicate that CD32 expression in memory CD4 T cells from blood and LNs is not restricted to HIV-1-infected individuals and that CD32^+^ CD4 T cells are greatly enriched in LNs.

### Distribution of CD32 in LN and blood memory CD4 T cell populations.

**FIG 2:**
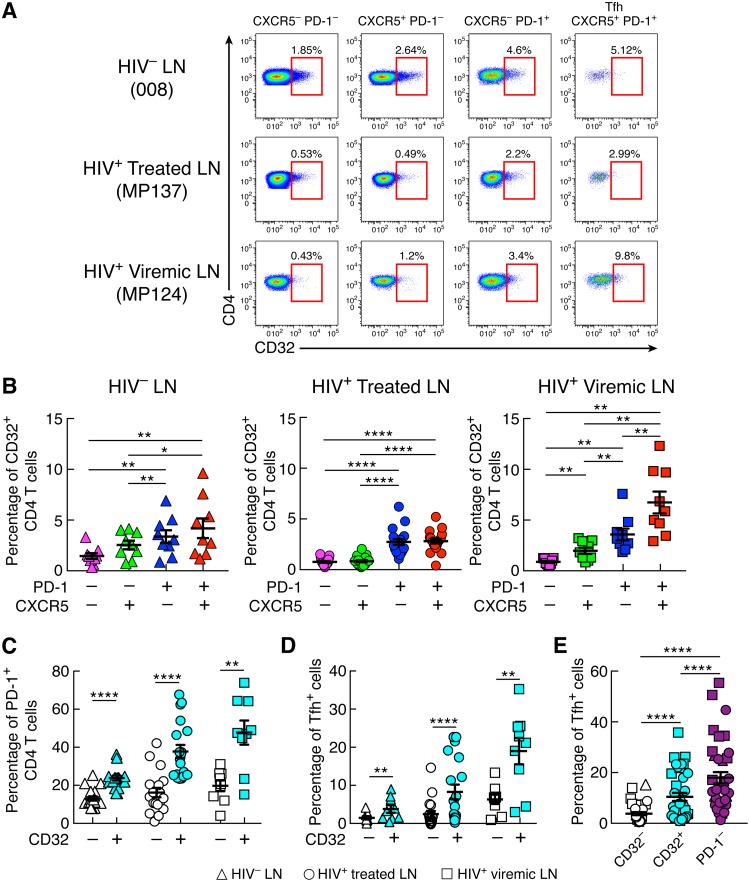
CD32^+^ CD4 T cells from LNs are enriched in PD-1^+^ and Tfh cells. (A) Representative flow cytometry profiles of CD32 expression in LN memory CD4 T cell populations defined by PD-1 and CXCR5 expression in HIV-1-uninfected and HIV-1-infected ART-treated and viremic individuals. (B) Cumulative data on the frequencies of CD32^+^ memory CD4 T cells within PD-1^−^ CXCR5^−^, PD-1^−^ CXCR5^+^, PD-1^+^ CXCR5^−^, and Tfh (PD-1^+^ CXCR5^+^) cell populations isolated from LNs of 9 HIV-1-uninfected, 19 ART-treated, and 9 viremic individuals. (C) Percentages of PD-1^+^ cells in gated CD32^−^ and CD32^+^ memory (CD45RA^−^) CD4 T cell populations in the three groups. (D) Distribution of Tfh cells within CD32^−^ and CD32^+^ memory CD4 T cell populations. (E) Distribution of Tfh cells within CD32^−^, CD32^+^, and PD-1^+^ CD4 T cell populations from lymph nodes. *, *P* < 0.05; **, *P* < 0.01; ****, *P* < 0.0001 (values were obtained by a Wilcoxon signed-rank test, and error bars denote means ± SEM).

**FIG 3:**
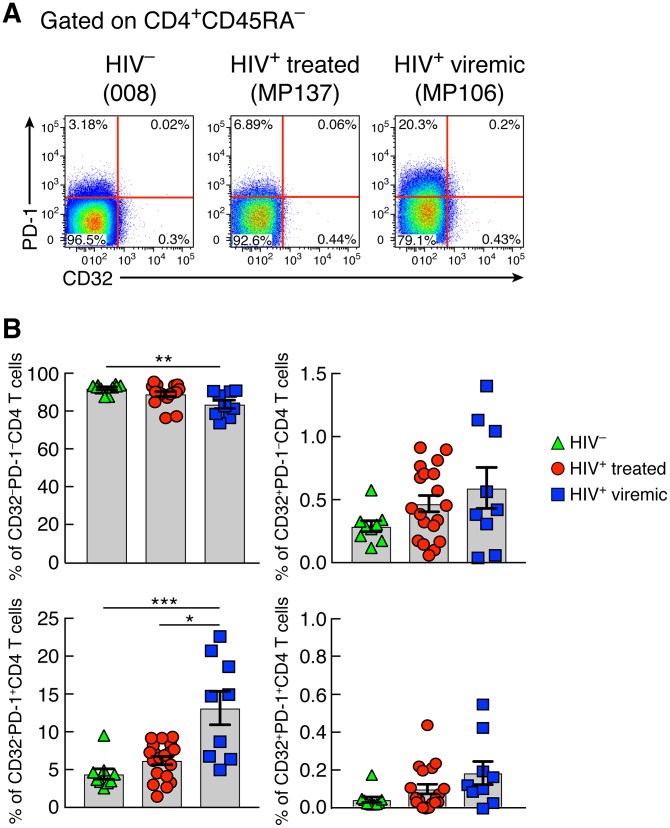
Frequencies of blood memory CD4 T cell populations on the basis of CD32 and PD-1. (A) Representative flow cytometry profiles of blood memory (CD45RA^−^) CD4 T cell populations expressing CD32 and/or PD-1 from representative HIV-1-uninfected, ART-treated, and viremic individuals. (B) Cumulative data on the frequencies of CD32^−^ PD-1^−^, CD32^+^ PD-1^−^, CD32^−^ PD-1^+^, and CD32^+^ PD-1^+^ CD4 T cell populations in blood mononuclear cells from HIV-1-uninfected (*n* = 9) and HIV-1-infected ART-treated (*n* = 19) and viremic (*n* = 9) individuals. **, *P* < 0.01; ***, *P* < 0.001 (values were obtained by a Mann-Whitney test, and error bars denote means ± SEM).

Next, we investigated the distribution of CD32 in different memory LN CD4 T cell populations defined by the expression of CXCR5 and PD-1 in HIV-uninfected, long-term ART-treated, and viremic individuals. Memory CD32^+^ CD4 T cells were consistently enriched in PD-1^+^ CXCR5^−^ and PD-1^+^ CXCR5^+^ CD4 T cell populations in the three study groups (*P* < 0.05 for all study groups) (Fig. 2A and B). The PD-1^+^ CXCR5^+^ CD4 T cells correspond to Tfh cells (12, 18–20). PD-1^+^ CD4 T cells were greatly enriched within the CD32^+^ CD4 T cell population in the three study groups, and 40 and 50% of PD-1^+^ CD4 T cells coexpressed PD-1 and CD32 in long-term ART-treated and viremic individuals, respectively (Fig. 2C) (*P* < 0.0001 for HIV-uninfected and ART-treated individuals and *P* = 0.003 for viremics). Similarly, substantial proportions of Tfh cells, ranging between 10% in ART-treated individuals and 20% in viremic individuals, were contained within the CD32^+^ CD4 T cell population (Fig. 2D) (*P* < 0.0001 and *P* = 0.003, respectively). However, compared to the CD32^+^ CD4 T cell population, Tfh cells were significantly enriched within the PD-1^+^ CD4 T cell population (*P* < 0.0001) (Fig. 2E). The distribution of memory CD4 T cell populations defined by CD32 and PD-1 was also investigated in blood (Fig. 3A and B). The CD32^−^ PD-1^−^ CD4 T cell population was significantly reduced in viremic individuals compared to HIV-uninfected subjects (*P* = 0.002). No differences in the percentages of CD32^+^ PD-1^−^ and CD32^+^ PD-1^+^ CD4 T cells were observed in the three study groups, while percentages of CD32^−^ PD-1^+^ CD4 T cells were significantly increased in viremic individuals (13.1% in viremics versus 4.3% in HIV-uninfected individuals [*P* = 0.0005] and 6.1% in treated individuals [*P* = 0.01]).

### Relationship between CD32^+^ and PD-1^+^ CD4 T cell populations.

**FIG 4:**
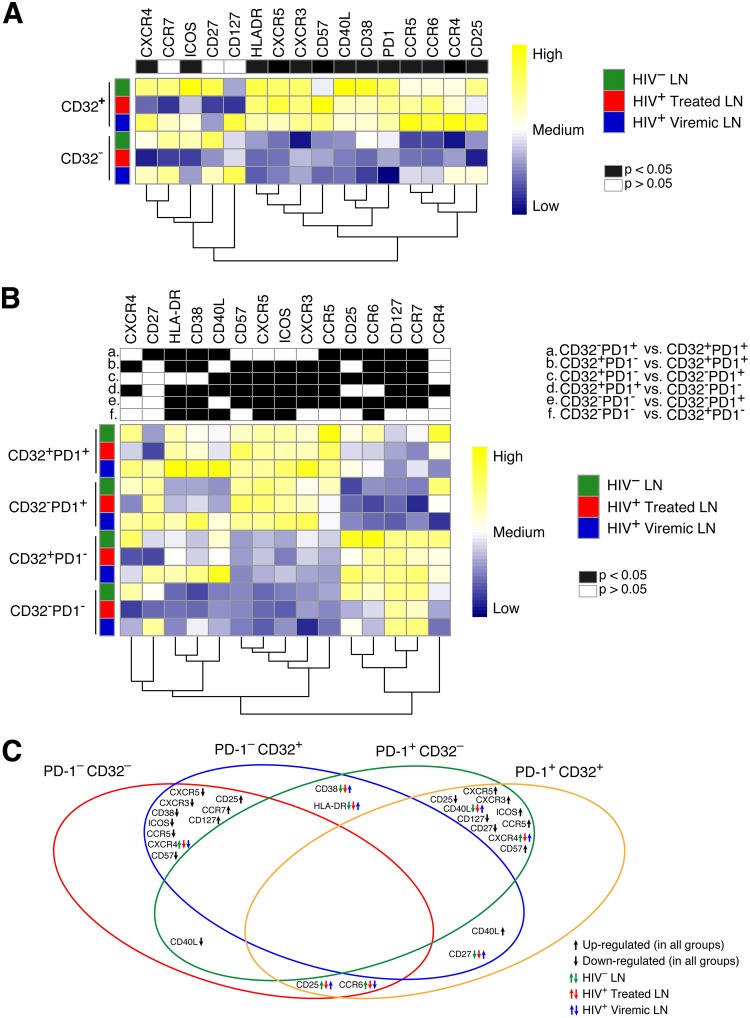
Mass cytometry analysis of LN memory CD4 T cells defined by CD32 and/or PD-1 expression. Mass cytometry staining was performed on LN mononuclear cells isolated from 9 HIV-1-uninfected, 16 HIV-1-infected ART-treated, and 9 viremic individuals. Cells were stained with a panel of 30 cell surface markers (Table 2). (A and B) Heat maps of mean marker expression levels in memory CD4 T cells defined on the basis of CD32 (A) and CD32 and PD-1 (B) expression. Columns (i.e., markers) are scaled to facilitate comparisons of expression values color-coded from blue (low) to yellow (high). (C) Venn diagram summarizing common trends and the direction of changes for significant markers (as in panel B) in CD45RA^−^ CD4 T cells defined on the basis of PD-1 and CD32 expression. Black arrows indicate trends that are common to all study groups (HIV-uninfected, ART-treated, and viremic individuals). Green, red, and blue arrows indicate trends that are specific to each study group.

The simultaneous assessment by mass cytometry of 30 phenotypic markers defining different cell lineages, memory differentiation, cell activation, and cell trafficking has provided the possibility to analyze the differences between CD32^+^ and CD32^−^ lymph node memory CD4 T cell populations in the three study groups in more depth. The levels of the large majority (13 out of 16) of markers defining chemokine receptors (CXCR3, CCR6, and CCR4), cell activation (CD25, CD38, and HLA-DR), memory cell differentiation (ICOS, CD57, CD27, CD127, CXCR5, PD-1, and CD40L), and HIV-1 coreceptors (CCR5 and CXCR4) were greatly increased in CD32^+^ compared to CD32^−^ CD4 T cells regardless of the study group (Fig. 4A). Only CCR7, CD127, and CD27 were unchanged. The heat maps in Fig. 4A also show the differences among the study groups. The levels of ICOS, CD38, and PD-1 were enhanced within CD32^+^ cells (*P* < 0.0001), while the levels of CCR4 and CD127 were slightly reduced within CD32^−^ cells in viremic individuals. Several markers were downregulated in ART-treated individuals compared even to HIV-uninfected individuals.

Due to the overlap between CD32^+^ and PD-1^+^ CD4 T cell populations, the distribution of the same panel of makers was analyzed in lymph node memory CD4 cell subpopulations defined by the expression of CD32 and PD-1 in the three study groups (Fig. 4B). The heat maps and the Venn diagram show that the expression of PD-1 determines a clear separation between the four cell populations. It appears that PD-1^−^ CD32^−^ and PD-1^−^ CD32^+^ CD4 T cells on one side and PD-1^+^ CD32^−^ and PD-1^+^ CD32^+^ CD4 T cells on the other side share similar phenotypes and are closely related to each other (Fig. 4B and C). The higher expression levels of markers such as CXCR5, ICOS, CD57, CD40L, CD38, and HLA-DR and reduced expression levels of CD127 and CCR7 indicate that PD-1^+^ CD4 T cell populations are at an advanced stage of differentiation and activation. Of note, the expression of the HIV-1 coreceptors CCR5 and CXCR4 is greatly enhanced in the PD-1^+^ CD4 T cell populations (for CCR5^+^ cells, 21.8% in CD32^−^ PD-1^+^ and 37% in CD32^+^ PD-1^+^ populations; for CXCR4^+^ cells, 14.2% in CD32^−^ PD-1^+^ and 18% in CD32^+^ PD-1^+^ populations).

Taken together, these results indicate that there is great overlap between CD32^+^ and PD-1^+^ memory CD4 T cell populations in LNs of HIV-1-infected individuals. Importantly, the expression of PD-1 independently of CD32 expression helps to define CD4 T cells that are potentially more susceptible to HIV infection.

### Role of PD-1^+^ and CD32^+^ memory CD4 T cell populations in the HIV reservoir.

**FIG 5:**
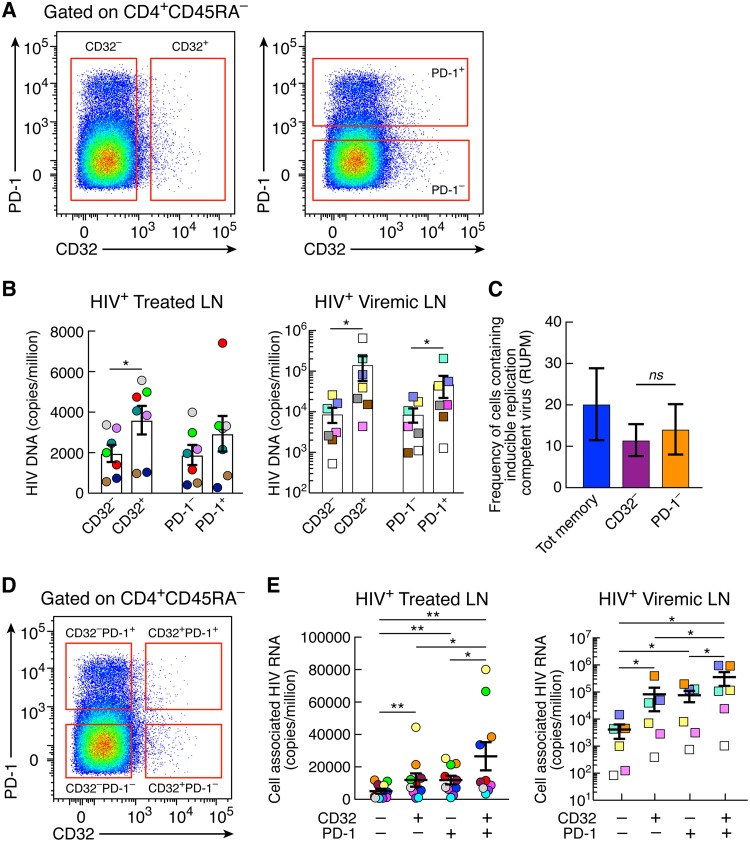
Levels of total HIV DNA and cell-associated HIV RNA in CD32^+^ and PD-1^+^ CD4 T cell populations. (A) Gating strategy for CD32^+^ and CD32^−^ and PD-1^+^ and PD-1^−^ memory (CD45RA^−^) CD4 T cell populations. (B) Quantification of total HIV DNA (copies per million cells) of sorted memory (CD45RA^−^) CD4 T cells expressing or not expressing CD32 and PD-1 from 7 aviremic ART-treated (round circles) and 7 viremic (squares) individuals. (C) Frequencies of cells with inducible replication-competent HIV in sorted CD32^−^, PD-1^−^, and total memory CD4 T cells estimated using conventional limiting-dilution methods by extreme limiting-dilution analysis (http://bioinf.wehi.edu.au/software/elda/) in 3 aviremic ART-treated individuals. (D) Gating strategy for CD32^−^ PD-1^−^, CD32^+^ PD-1^−^, CD32^−^ PD-1^+^, and CD32^+^ PD-1^+^ memory (CD45RA^−^) CD4 T cell populations. (E) Levels of cell-associated unspliced HIV RNA (copies/million cells) in sorted CD32^−^ PD-1^−^, CD32^+^ PD-1^−^, CD32^−^ PD-1^+^, and CD32^+^ PD-1^+^ memory CD4 T cell populations isolated from 10 aviremic ART-treated and 6 viremic individuals. *, *P* < 0.05; **, *P* < 0.01; ns, not significant (values were obtained by a Wilcoxon signed-rank test, and error bars denote means ± SEM).

Having defined the distribution and the phenotypic relationship of PD-1^+^ and CD32^+^ memory CD4 T cell populations in blood and LNs, we sought to investigate the role of these cell populations in the HIV reservoir through determining whether there is (i) an enrichment of HIV DNA-containing cells in LN CD32^+^ and PD-1^+^ CD4 T cells, (ii) a difference in the frequencies of HIV DNA-containing cells between LN CD32^+^ and PD-1^+^ CD4 cells in viremic and long-term ART-treated individuals, (iii) a difference in the frequencies of cells containing replication-competent HIV between LN CD32^+^ and PD-1^+^ CD4 T cells in long-term-treated individuals, and (iv) a difference in HIV transcription between LN CD32^+^ and PD-1^+^ CD4 T cells in long-term ART-treated individuals. Memory CD4 T cells (CD45RA^−^) isolated from lymph nodes of seven long-term ART-treated and seven viremic individuals were sorted on the basis of CD32 and/or PD-1 expression and analyzed for the presence of total HIV DNA. A caveat of this analysis is that because of the overlap between CD32^+^ and PD-1^+^ CD4 T cell populations, a proportion of cells coexpressing PD-1 and CD32 is present in both sorted CD32^+^ and PD-1^+^ cell populations (Fig. 5A). However, because of the limited number of cells, particularly in LNs of ART-treated individuals, it was not possible to assess the total HIV DNA in the four cell populations defined by the expression of CD32 and PD-1 (CD32^−^ PD-1^−^, CD32^+^ PD-1^−^, CD32^−^ PD-1^+^, and CD32^+^ PD-1^+^). CD32^+^ and PD-1^+^ CD4 T cell populations were enriched in cells containing HIV DNA compared to CD32^−^ and PD-1^−^ cell populations. With regard to CD32^+^ versus CD32^−^ populations, the differences were significant in the ART-treated individuals (1.83-fold; *P* = 0.01) and viremics (23.7-fold; *P* = 0.01) (Fig. 5B). With regard to PD-1^+^ versus PD-1^−^ CD4 T cell populations, there was only a trend toward higher HIV DNA in PD-1^+^ versus PD-1^−^ populations in ART-treated individuals, while the differences were significant in viremic individuals (6.5-fold; *P* = 0.01) (Fig. 5B).

These results show an enrichment of HIV-infected cells within CD32^+^ and PD-1^+^ LN memory CD4 T cells but not a substantial difference between these cell populations. The massive proportions of cells infected on the basis of the total HIV DNA content in CD32^+^ and PD-1^+^ CD4 T cells are consistent with the phenotypic data (Fig. 4) showing increased expression of HIV-1 coreceptors in these cell populations and indicate that they may serve as a major reservoir of HIV-1.

To estimate the frequencies of HIV-1-infected cells containing inducible replication-competent virus, a quantitative virus outgrowth assay (QVOA) was performed on three ART-treated individuals. The objective of the QVOA was to determine whether the exclusion of CD32^+^ or PD-1^+^ cells had a different impact on the frequency of HIV-1-infected cells with inducible virus. Because of the limited number of cells isolated from ART-treated individuals, it was not possible to perform the QVOA on CD32^+^ and PD-1^+^ CD4 T cell populations. For the QVOA, different cell concentrations (5-fold limiting dilutions of 10^5^, 2 × 10^4^, and 4 × 10^3^) and multiple replicates of sorted viable lymph node CD32^−^, PD-1^−^, and total memory (CD45RA^−^) CD4 T cells (Fig. 5A) were cocultured with allogeneic fresh CD8 T cell-depleted blood mononuclear cells from HIV-1-uninfected individuals, and frequencies were estimated at day 14 using an extreme limiting-dilution assay (13, 21, 22). These analyses provide the average RNA units per million (RUPM) for each cell population investigated. The frequencies of cells with inducible HIV RNA in CD32^−^ and PD-1^−^ CD4 cell populations were not significantly different (Fig. 5C). In order to interpret these results correctly, it is important to consider that by sorting out CD32^+^ cells, there are still PD-1^+^ cells remaining in the sorted CD32^−^ cell population (Fig. 5A). Since PD-1^+^ cells also contain inducible HIV, it is expected that there is a trend toward a reduction of the frequency of cells with inducible HIV and not a more dramatic reduction. Along the same line, by sorting out PD-1^+^ cells, there are still CD32^+^ cells remaining in the sorted PD-1^−^ cell population, which explains the partial reduction found. Finally, there was a trend toward higher frequencies of cells with inducible HIV RNA in the total memory CD4 T cell population than in the CD32^−^ and PD-1^−^ populations (Fig. 5C).

We have recently shown that PD-1^+^ CD4 T cells serve as the major CD4 T cell compartment in blood and LNs for replication-competent and infectious HIV-1 and for active and persistent virus transcription in long-term ART-treated aviremic individuals (13). We then investigated whether there was a difference in HIV transcription between LN CD32^+^ and PD-1^+^ CD4 T cells in 10 long-term ART-treated and 6 viremic individuals. To determine the cell compartment(s) serving as the site of active and persistent HIV-1 transcription, cell-associated HIV-1 RNA was assessed in LN memory CD32^−^ PD-1^−^, CD32^+^ PD-1^−^, CD32^−^ PD-1^+^, and CD32^+^ PD-1^+^ CD4 T cell populations. The gating strategy for cell sorting is shown in Fig. 5D. The results indicate that the levels of cell-associated HIV-1 RNA were significantly higher in CD32^+^ PD-1^+^ CD4 T cells than in the other three cell populations (Fig. 5E). CD32^+^ PD-1^+^ CD4 T cells were enriched in cell-associated HIV-1 RNA compared to CD32^−^ PD-1^−^ (averages of 5.2-fold in ART-treated individuals and 86.6-fold in viremics), CD32^+^ PD-1^−^ (2.2-fold in ART-treated individuals and 4.3-fold in viremics), and CD32^−^ PD-1^+^ (averages of 2.2-fold in ART-treated individuals and 4.6-fold in viremics) cell populations. CD32^+^ PD-1^−^ and CD32^−^ PD-1^+^ cell populations also had significantly higher levels of cell-associated HIV RNA than CD32^−^ PD-1^−^ cells (*P* < 0.05), while no differences were found between CD32^+^ PD-1^−^ and CD32^−^ PD-1^+^ cell populations.

Therefore, the coexpression of CD32 and PD-1 identifies a population with greater HIV transcriptional activity within Tfh cells, and the sole expression of CD32 identifies a cell population with levels of transcriptional activity similar to those measured in single PD-1^+^ CD4 T cells.

Recent results (23) have suggested that the expression of CD32 on blood CD4^+^ T cells was reflective of either the presence of doublets or partial exchange of B cell and CD4^+^ T cell membranes. We have also carefully addressed this issue in our flow cytometry experiments. The exclusion of doublets is a mandatory step in the flow cytometry analysis of cell populations. In the present study, we have totally excluded that the expression of CD32 on lymph node CD4^+^ T cells was the result of B-T cell doublets. In the presence of B-CD4 T cell doublets, we should find that the expression of CD32 in the different memory CD4 T cell populations would also be associated with the expression of CD19. The doublets have been excluded in two sequential analyses. Similarly, CD19^+^ B cells and CD8^+^ T cells have been excluded when gating for CD4^+^ T cells, and we have no evidence of CD19 expression on single CD32^+^ CD4 T cells as well as CD32^+^ PD-1^+^ CD4 T cells.

### CD32^+^ and PD-1^+^ CD4 T cells and markers of HIV disease activity.

**FIG 6:**
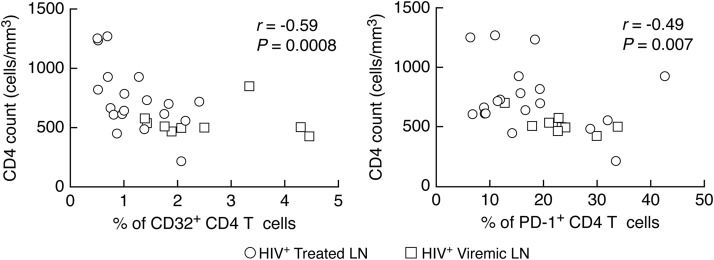
Correlations between memory CD4 T cell populations defined by CD32 and PD-1 CD4 counts. Shown are correlations between the percentages of CD32^+^ and PD-1^+^ memory CD4 T cells and CD4 T cell counts. A Spearman rank test was used for correlations.

**FIG 7:**
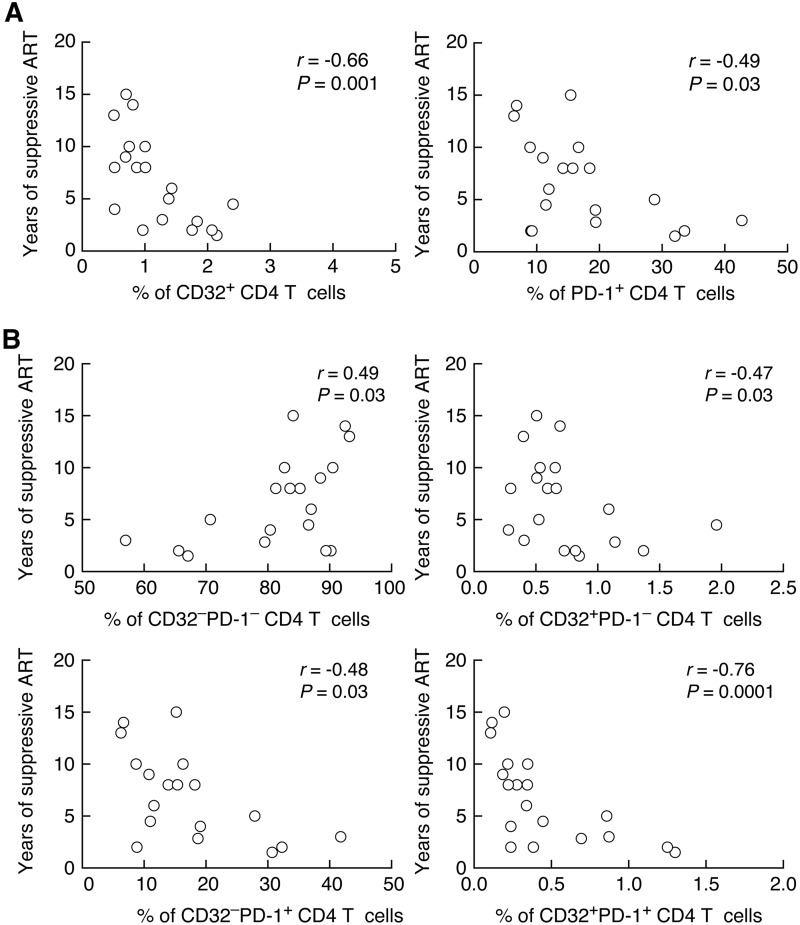
Correlation between memory CD4 T cell populations defined by CD32 and/or PD-1 and years of suppressive ART. (A) Correlation between the percentages of CD32^+^ and PD-1^+^ memory CD4 T cells and years of suppressive ART. (B) Correlation between the percentages of CD32^−^ PD-1^−^, CD32^+^ PD-1^−^, CD32^−^ PD-1^+^, and CD32^+^ PD-1^+^ populations and years of suppressive ART. A Spearman rank test was used for correlations.

We then determined the relationship between CD32^+^ and PD-1^+^ CD4 T cells and indicators of HIV disease activity and the length of suppressive ART. The percentage of CD32^+^ and PD-1^+^ CD4 T cells negatively correlated with the CD4 T cell count (*r* = −0.59 and *P* = 0.0008 and *r* = −0.49 and *P* = 0.007, respectively) (Fig. 6) and length of ART treatment (*r* = −0.66 and *P* = 0.001 and *r* = −0.49 and *P* = 0.03, respectively) (Fig. 7A). Next, we analyzed the relationships between the length of treatment and the percentages of the four LN memory CD4 T cell populations gated on the basis of the expression of CD32 and PD-1. Interestingly, the percentage of CD32^−^ PD-1^−^ cells positively correlated with the length of ART (*r* = 0.49 and *P* = 0.03), while the percentages of CD32^+^ PD-1^−^, CD32^−^ PD-1^+^, and CD32^+^ PD-1^+^ cells negatively correlated with the length of ART treatment (*r* = −0.47 and *P* = 0.03, *r* = −0.48 and *P* = 0.03, and *r* = −0.76 and *P* = 0.0001, respectively) (Fig. 7B).

These results indicate that the expansion of both CD32^+^ and PD-1^+^ memory CD4 T cell populations in lymph nodes correlates with markers of HIV disease activity.

## DISCUSSION

The percentage (range of 0.2 to 0.5%) of CD32^+^ CD4 T cells and their intensity of expression are extremely low in blood, thus rendering in-depth characterization of circulating CD32^+^ CD4 T cells challenging. For these reasons, we decided to study CD32^+^ CD4 T cells in LNs. Lymphoid tissues are the major anatomic compartment for HIV replication, spreading, and persistence, and therefore, they are the ideal anatomic compartment to understand the role of CD32^+^ CD4 T cells in the HIV reservoir and their relationship with PD-1^+^/Tfh cells that have been shown to serve as a major reservoir for HIV (13, 24–31). We have previously demonstrated that PD-1^+^/Tfh cell populations serve as a major reservoir for HIV-1 in both viremic and long-term ART-treated subjects (12, 13). These observations are instrumental since they indicate that the expression of PD-1 is a marker to define the dominant CD4 T cell population infected with HIV-1 and containing replication-competent virus. Along the same line, both viremic and long-term-treated subjects have been studied to better understand the role of CD32 in defining cell populations serving as a reservoir for HIV-1. The use of both groups of patients is critical to provide insights into the kinetics of the HIV reservoir following initiation of therapy and the changes occurring in the cell populations defined by the expression of PD-1 and CD32.

The percentages of CD32^+^ CD4 T cells in the blood were similar in HIV-uninfected, viremic, and ART-treated individuals. Higher percentages of CD32^+^ CD4 T cells were found in LNs in the three study groups and were significantly expanded in viremic individuals, but similar percentages were found in LNs of HIV-uninfected and treated individuals, thus indicating that CD32 expression on CD4 T cells is not exclusive of HIV infection, as proposed by Descours et al. (14).

CD32^+^ and PD-1^+^ CD4 T cell populations are tightly correlated, as indicated by the large proportion (up to 50%) of CD32^+^ CD4 T cells coexpressing PD-1. The coexpression of PD-1 is important for understanding the status of activation and differentiation of CD32^+^ cells and their role in the HIV reservoir. Indeed, the expression of PD-1 together with HLA-DR and CD38 in CD32^−^ PD-1^+^ and CD32^+^ PD-1^+^ CD4 T cells indicates that these cell populations are associated with high levels of cell activation and advanced differentiation compared to the CD32^−^ PD-1^−^ and CD32^+^ PD-1^−^ cell populations. However, the CD32^+^ PD-1^−^ cell population defines a population of cells expressing higher levels of CD25, CCR6, and CCR4. In addition, there are also increased expression levels of CD127 and CCR7. Differently from PD-1^+^ cells, this population expresses lower levels of CD57, CXCR5, ICOS, CXCR3, and CCR5. Therefore, CD32^+^ PD1^−^ cells represent a distinct population different from PD-1^+^ cells and, at least in part, the double-negative cell population (CD32^−^ PD-1^−^).

Of note, CD32^+^ and PD-1^+^ CD4 T cells expressed higher levels of the HIV-1 coreceptors CCR5 and CXCR4, thus rendering these cells a preferential target for HIV infection. These data are consistent with two recent studies (15, 16) showing that CD32^+^ CD4 T cells express higher levels of activation markers and HIV coreceptors.

On the basis of the quantification of the total HIV DNA and of cells with inducible replication-competent HIV, CD32^+^ and PD-1^+^ CD4 T cells contribute equally to the HIV reservoir in long-term-treated individuals, and there were also no differences found in viremic individuals. More importantly, the analysis of cell-associated HIV RNA has demonstrated equal levels of persistent HIV transcription in CD32^+^ PD-1^−^ and CD32^−^ PD-1^+^ CD4 T cell populations and significantly higher levels of transcription in the CD32^+^ PD-1^+^ cells. These findings are overall in agreement with data from a recent study (15) that failed to confirm the conclusions of Descours et al. that CD32 defines the latent “elusive” HIV reservoir. However, the present study provides detailed information on the phenotypic differences between CD32^+^ PD-1^−^ and CD32^+^ PD-1^+^ lymph node CD4 T cell populations and a better delineation of the role of these distinct CD32^+^ cell populations in the HIV reservoir. The present results demonstrate that CD32^+^ CD4 T cells have high levels of HIV transcription. This finding is consistent with the observation that a large proportion of CD32^+^ CD4 T cells is activated. As mentioned above, it is important to underscore that about 50% of the CD32^+^ CD4 T cell population that coexpresses PD-1 and CD32^+^ PD-1^+^ lymph node CD4 T cells correspond to Tfh cells, which have been previously shown to serve as the major CD4 T cell HIV reservoir in both viremic and long-term ART-treated subjects (12, 13). However, CD32, independently of PD-1, defines an additional population of memory CD4 T cells with persistent HIV transcription. When coexpressed with PD-1, CD32 defines the CD4 T cell population with the highest levels of transcription.

In conclusion, the combined use of CD32 and PD-1 identify three cell populations, CD32^+^ PD-1^−^, CD32^−^ PD-1^+^, and CD32^+^ PD-1^+^, of memory CD4 T cells responsible for most of the persistent HIV transcription in long-term-treated individuals.

## MATERIALS AND METHODS

### Study groups.

Samples from 9 HIV-uninfected subjects, 19 subjects on suppressive ART, and 9 viremic individuals not receiving ART at the time of sampling were used in this study. None of the participants under ART had detectable plasma viremia at the time of the study, as assessed by viral load measurements using AmpliPrep/Cobas TaqMan HIV-1 test v 2.0 (Roche), with a detection limit of 20 HIV RNA copies/ml of plasma. All participants underwent lymph node biopsy (inguinal lymph nodes), and blood was collected at the same time. Subject characteristics are summarized in Table 1. These studies were approved by the Institutional Review Board of the Centre Hospitalier Universitaire Vaudois, and all subjects gave written informed consent.

### Cell isolation.

Inguinal lymph node biopsy specimens and blood samples were collected on the same day. Blood mononuclear cells were isolated as previously described (32), and lymph node mononuclear cells were isolated by mechanical disruption (24). Blood mononuclear cells and lymph node mononuclear cells were cryopreserved in liquid nitrogen.

### Antibodies.

The following antibodies were used: allophycocyanin (APC)-H7-conjugated anti-CD3 (clone SK7; BD), Pacific Blue (PB)-conjugated anti-CD19 (clone HIB19; BioLegend), PB-conjugated CD8 (clone RPA-T8; BD) fluorescein isothiocyanate (FITC)-conjugated anti-CXCR5 (clone RF8B2; BD), phycoerythrin (PE)-Cy7-conjugated anti-PD-1 (clone EH12.1; BD), Alexa 700 anti-CD4 (clone RPA-T4; BioLegend), APC-conjugated anti-CD32 (FUN-2; BioLegend), and phycoerythrin-Texas Red (ECD)-conjugated anti-CD45RA (clone 2H4; Beckman Coulter). An Aqua Live/Dead stain kit was used to determine the viability of cells. Cells were acquired on an LSRII flow cytometer using FACSDiva software (BD) and analyzed using FlowJo 9.7 (TreeStar Inc.) or sorted using FACSAria (BD). The CD32 antibody used in the present study is the same one used in the study by Descours et al., and this antibody does not discriminate between CD32a and CD32b. We used a fluorescence minus one (FMO) control to gate on CD32^+^ cells.

### CyTOF marker labeling and detection.

Cryopreserved lymph node mononuclear cells were thawed and resuspended in complete RPMI medium (Gibco, Life Technologies) (10% heat-inactivated fetal bovine serum [FBS] [Institut de Biotechnologies Jacques Boy], 100 IU/ml penicillin, and 100 μg/ml streptomycin [BioConcept]) at 1 × 10^6^ cells/ml.

Viability of cells in 500 μl of phosphate-buffered saline (PBS) was assessed by incubation with 50 μM cisplatin (Sigma-Aldrich) for 5 min at room temperature (RT), and then the reaction was quenched with 500 μl of fetal bovine serum. Next, cells were incubated for 20 min at 4°C with anti-CD32 APC antibody. Next, cells were washed and incubated for 30 min at RT with a 50-μl cocktail of cell surface metal-conjugated antibodies (Table 2). Cells were washed and fixed for 10 min at RT with 2.4% paraformaldehyde (PFA). Total cells were identified by DNA intercalation (1 μM Cell-ID intercalator; Fluidigm/DVS Science) in 2% PFA at 4°C overnight. Labeled samples were assessed by using a CyTOF1 instrument that was upgraded to CyTOF2 (Fluidigm), using a flow rate of 0.045 ml/min. Flow cytometry standard (FCS) files were normalized to EQ Four Element calibration beads using CyTOF software. For conventional cytometric analysis of memory CD4 T cell populations, FCS files were imported into Cytobank data analysis software.

**TABLE 2:** Mass cytometry panel

Target	Metal	Company	Clone
CD4	^115^In	BioLegend	RPA-T4
CCR6	^141^Pr	Fluidigm/DVS	G034E3
CD19	^142^Nd	Fluidigm/DVS	HIB19
ICOS	^143^Nd	BioLegend	C398.4A
CD8	^145^Nd	BioLegend	RPA-T8
IgD	^146^Nd	Fluidigm/DVS	IA6-2
CD7	^147^Sm	Fluidigm/DVS	CD7-6B7
CD57	^148^Nd	BD	G10F5
CCR4	^149^Sm	Fluidigm/DVS	205410
CXCR5	^153^Eu	Fluidigm/DVS	RF8B2
CD21	^152^Sm	Fluidigm/DVS	BL13
CXCR3	^154^Sm	BioLegend	G025H7
CD27	^155^Gd	Fluidigm/DVS	L128
CD11c	^156^Gd	BioLegend	3.9
CCR7	^159^Tb	Fluidigm/DVS	G043H7
CD25	^158^Gd	BioLegend	M-A251
CD14	^160^Gd	Fluidigm/DVS	M5E2
CD1C	^161^Dy	BioLegend	L161
CD32-APC	^162^Dy	Fluidigm/DVS	FUN2
CD20	^166^Er	BioLegend	2H7
CD38	^167^Er	Fluidigm/DVS	HIT2
CD45RA	^169^Tm	Fluidigm/DVS	HI100
CD40L	^168^Er	Fluidigm/DVS	CD40L
CD3	^170^Er	Fluidigm/DVS	UCHT1
CCR5	^171^Yb	Fluidigm/DVS	NP-6G4
HLA-DR	^173^Yb	Fluidigm/DVS	L243
PD-1	^174^Yb	Fluidigm/DVS	EH12.2H7
CXCR4	^175^Lu	Fluidigm/DVS	12G5
CD127	^176^Yb	Fluidigm/DVS	A019D5
CD16	^209^Bl	Fluidigm/DVS	3G8
Live/Dead	^195^Pt	Fluidigm/DVS	Cell-ID

### Quantification of total HIV DNA.

CD32^+^, CD32^−^, PD-1^+^, and PD-1^−^ memory (CD45RA^−^) CD4 T cell populations were sorted using a FACSAria instrument, and lysates were directly used in a nested PCR to quantify both total HIV DNA and CD3 gene copy numbers, as previously described (7).

### Quantification of cell-associated RNA.

Cell-associated HIV-1 RNA (unspliced HIV RNA encoding the region between the 5' long terminal repeats [5′-LTRs] and *gag*) from individual samples was quantified from CD4 T cell populations sorted on the basis of CD32 and PD-1 expression (CD32^−^ PD-1^−^, CD32^+^ PD-1^−^, CD32^−^ PD-1^+^, and CD32^+^ PD-1^+^) and subjected to DNase treatment (RNAqueous-4PCR kit; Ambion) (33). RNA standard curves were generated after isolation and quantification of viral RNA from the supernatant of an ACH2 culture as previously described (33). One-step cDNA synthesis and preamplification were performed as previously described (34).

### Quantitative viral outgrowth assay.

Different cell concentrations (5-fold limiting dilutions of 10^5^, 2 × 10^4^, and 4 × 10^3^ for lymph node CD4 T cells) and five replicates of sorted viable LN total memory (CD45RA^−^) and CD32^−^ and PD-1^−^ memory CD4 T cells isolated from three ART-treated HIV-1-infected individuals were cultured with allogeneic fresh CD8^−^-depleted blood mononuclear cells (10^6^ cells/ml) from HIV-1-uninfected subjects in the presence of anti-CD3/anti-CD28 monoclonal antibody (MAb)-coated plates (10 μg/ml) for 3 days (13, 22). Cells were carefully transferred to new uncoated plates after 3 days of activation. Cells under all conditions were cultured in complete RPMI for 14 days. Medium was replaced at day 5. Supernatants were collected at day 14. The presence of HIV-1 RNA was assessed by a Cobas AmpliPrep/TaqMan HIV-1 test (Roche, Switzerland). Wells with detectable HIV-1 RNA (≥20 HIV-1 RNA copies/ml) were referred to as HIV-1 RNA-positive wells. RUPM frequencies were estimated by conventional limiting-dilution methods using extreme limiting-dilution analysis (http://bioinf.wehi.edu.au/software/elda/) (21).

### Statistical analysis.

We performed a two-tailed Mann-Whitney test to compare frequencies of CD32^+^ CD4 T cells among HIV-1-uninfected and HIV-1-infected ART-treated and viremic individuals. A Wilcoxon signed-rank test was used to compare different subsets. Correlative analyses were done using a Spearman test. These analyses were done using Prism 7.0.

Statistical analyses comparing cell surface marker expression levels (nontransformed) (Fig. 4A and B) in CD32^+^ CD4 T cells and subsets defined by CD32 and PD-1 (i.e., CD32 versus PD-1 quadrants) were assessed using linear mixed-effect models on the frequencies of positive cells for each marker accounting for differences between patient groups (HIV-1-uninfected, HIV-1-infected ART-treated, and viremic individuals) with patient-level random intercepts. *P* values were adjusted using Bonferroni correction, and data analysis was performed using R statistical software and the lmer package.

### Data availability.

All relevant data are within the paper.
